# A fast and adaptable method for high accuracy integration of the time-dependent Schrödinger equation

**DOI:** 10.1038/s41598-018-37382-0

**Published:** 2019-01-28

**Authors:** Daniel Wells, Harry Quiney

**Affiliations:** ARC Centre of Excellence for Advanced Molecular Imaging, Theoretical Condensed Matter Physics Group, School of Physics, University of Melbourne, Victoria, 3010 Australia

## Abstract

We present an adaptable, fast, and robust method for integrating the time-dependent Schrödinger equation. We apply the method to calculations of High Harmonic (HHG) and Above Threshold Ionisation (ATI) spectra for a single atomic electron in an intense laser field. Our approach implements the stabilized bi-conjugate gradient method (BiCG-STAB) for solving a sparse linear system to evolve the electronic wavefunction in time. The use of this established method makes the propagation scheme less restrictive compared to other schemes which may have particular requirements for the form of the equation, such as use of a three-point finite-difference approximation for spatial derivatives. Our method produces converged solutions significantly faster than existing methods, particularly if high accuracy is required. We demonstrate that this approach is suitable for a range of different parameters and show that in many circumstances significant gains can be made with the use of a fourth-order time propagator as opposed to the more common second-order Crank-Nicolson (CN) method.

## Introduction

There is substantial interest in using the interaction of atoms in intense laser fields as a source of coherent short-wavelength light^1–5^. Such light sources are attractive for their laboratory scale and are the foundation of attosecond science, which exploits the sub-cycle dynamics of electron behaviour to probe matter directly on its natural timescale^6–9^.

Numerical integration of the Time-Dependent Schrödinger Equation (TDSE) has been an essential tool in understanding intense laser-atom interactions since practical applications of this field of research became apparent with the discoveries of Above Threshold Ionization (ATI)^10^ and High Harmonic Generation (HHG)^11^. Direct solution of the TDSE provides an *ab initio* treatment of the interaction capable of dealing exactly with its non-linear nature, which has historically been exploited to obtain insights that would not be possible with analytic or approximate methods^12^.

Despite the centrality of the TDSE to this field, high accuracy computations are rarely performed. This is due in part to the substantial computational requirements of such a task, but also due to the difficulty in directly comparing the results of numerical simulation with experimental observables. This is a result of both uncertainty in the precise form of the laser field and the necessity of making approximations in treating many electron atoms.

Interest in accurate numerical data, however, has increased recently largely due to developments in laser science that allow for the creation of tailored laser fields^13^ as well as experiments using atomic hydrogen as the interaction medium — a system for which the single particle Hamiltonian can be expressed exactly — and the use of such experiments for laser calibration^14–16^.

Although established techniques for integrating the TDSE^17–23^ can, in principle, work to arbitrary accuracy, they are not generally amenable to high accuracy simulations since computation time scales poorly with increased demand for accuracy. The commonly used split-step operator method^19,20^ separates the Hamiltonian into a field-free (atomic) term and an interaction (laser) term and uses a second-order decomposition of the time propagator,

1 $$U(t+{\rm{\Delta }}t;t)={e}^{-i{H}_{0}{\rm{\Delta }}t/2}{e}^{-i{H}_{{int}}{\rm{\Delta }}t}{e}^{-i{H}_{0}{\rm{\Delta }}t/2}+O({\rm{\Delta }}{t}^{3}),$$

which has an error proportional to the second-order commutator of the two terms that can be large at small radial distances^22^. Higher-order split operator methods have been developed based on higher-order factorisations of the exponential propagator^24–28^ and have been shown to be superior to the second-order method. Fundamentally however, these methods all rely on a division of the Hamiltonian, which adds complexities to the propagation such as the requirement for negative or complex time steps at high orders. The method we propose is simple and general in that it treats the Hamiltonian directly, eliminating the need for transformations between bases. Spectral methods^17^, while accurate in the sense that they compute the momentum operator to all orders, are computationally restrictive due to reliance on the fast Fourier transform.

An alternative to split operator methods, based directly on the Crank-Nicolson (CN) propagator, is the Matrix Iterative (MI) scheme^22^, which performs the matrix inversion by an iterative procedure that switches between the off-diagonal and diagonal parts of the Hamiltonian. Although this method has the advantage of avoiding operator splitting, it requires the direct solution of the atomic part of the propagator, which places restrictions on the form of the propagator and its discretization. Specifically, to be efficiently computed, the atomic Hamiltonian must be represented by nothing more complex than a tridiagonal matrix. In practice, this means the spatial derivatives must be represented by a three-point difference formula. We demonstrate that this is a clear disadvantage and significantly increases the number of grid points needed to meet a given accuracy. Similarly, MI uses the second-order CN method, which places a strong upper limit on the size of the time step that can be taken. For simulations where the interaction term is very large, this can lead to an unacceptable loss of accuracy making high accuracy simulations a demanding task.

The difficulty in implementing implicit schemes with higher-order spatial and temporal derivatives is that matrix inversion becomes inefficient by direct means. We show, however, that a general approach to solving the matrix equation that arises from implicit propagation schemes such as CN, based on the Stabilized Bi-Conjugate Gradient method (BiCG-STAB)^29^, enables efficient and accurate matrix inversion, but without the restrictions of a three-point spatial derivatives or a second-order time propagator. The success of the method depends on the presence of a suitable preconditioning matrix that, at least in some sense, approximates the propagating matrix and can be readily inverted. The BiCG-STAB scheme requires some additional computational overheads compared to established schemes but these are more than compensated for by its flexibility, in particular its ability to incorporate a five-point difference method for spatial derivatives.

## Methods

### Time propagation

The evolution of a quantum state is given by the time-dependent Schrödinger equation which, using atomic units units, is written

2 $$i\frac{d}{dt}{\rm{\Psi }}({\bf{r}},t)=H(t){\rm{\Psi }}({\bf{r}},t\mathrm{).}$$

For an initial state at time *t*, integration of this equation gives a state at time *t* + Δ*t*,

3 $${\rm{\Psi }}({\bf{r}},t+{\rm{\Delta }}t)={\mathscr{T}}\,\exp \,(\,-i\,{\int }_{t}^{t+{\rm{\Delta }}t}\,dtH(t))\,{\rm{\Psi }}({\bf{r}},t),$$

where ${\mathscr{T}}$ is the time-ordering operator.

Taking a short time approximation to this equation, in which the Hamiltonian is considered constant over a time step, and applying the Crank-Nicolson formula yields the familiar propagation equation

4 $$(1+i\frac{{\rm{\Delta }}t}{2}H){\rm{\Psi }}({\bf{r}},t+{\rm{\Delta }}t)=(1-i\frac{{\rm{\Delta }}t}{2}H)\,{\rm{\Psi }}({\bf{r}},t),$$

which is unitary, accurate to second-order in the time step and unconditionally stable.

Even for modest demands of accuracy, this equation necessitates a very small time step and so we also consider a higher-order expansion of equation 3, obtained from the fourth-order Lobatto IIIA which is an implicit Runge-Kutta method involving three function evaluations^30^. The dependence on the middle function evaluation can be removed by substitution to obtain:

5 $${U}^{(4)}(t+\frac{{\rm{\Delta }}t}{2};t+{\rm{\Delta }}t)\,{\rm{\Psi }}({\bf{r}},t+{\rm{\Delta }}t)={U}^{(4)}(t+\frac{{\rm{\Delta }}t}{2};t)\,{\rm{\Psi }}({\bf{r}},t),$$

6 $${U}^{(4)}(t^{\prime} ;t)=1-\frac{i(t^{\prime} -t)}{3}(2H(t^{\prime} )+H(t))-\frac{{(t^{\prime} -t)}^{2}}{3}H(t^{\prime} )H(t).$$

As an indication of the potential advantage of the higher-order method, consider the propagation of a plane wave with frequency *ω* such that

7 $${\rm{\Psi }}(t,{\bf{r}})={e}^{-i\omega t}\psi ({\bf{r}}\mathrm{).}$$

The wave function ${\rm{\Psi }}(t+{\rm{\Delta }}t,{\bf{r}})={e}^{-i\omega {\rm{\Delta }}t}{\rm{\Psi }}(t,{\bf{r}})$ is then approximated for the CN and fourth-order methods, respectively, by

8 $${\rm{\Psi }}(t+{\rm{\Delta }}t,{\bf{r}})=\frac{2-i\omega {\rm{\Delta }}t}{2+i\omega {\rm{\Delta }}t}{\rm{\Psi }}(t,{\bf{r}})$$

and

9 $${\rm{\Psi }}(t+{\rm{\Delta }}t,{\bf{r}})=\frac{12-6i\omega {\rm{\Delta }}t-{\omega }^{2}{\rm{\Delta }}{t}^{2}}{12+6i\omega {\rm{\Delta }}t-{\omega }^{2}{\rm{\Delta }}{t}^{2}}{\rm{\Psi }}(t,{\bf{r}}\mathrm{).}$$

The leading order error terms are (*ω*Δ*t*)^3^/12 and (*ω*Δ*t*)^5^/12000 respectively. Restricting this error to one part in one thousand gives *ω*Δ*t* < 0.23 for the second-order method and *ω*Δ*t* < 1.64 for the fourth-order method: the allowed time step is greater than a factor of seven larger. For a desired accuracy *ε* this factor of advantage of the higher-order method scales as *ε*^−2/15^.

The fourth-order method has an apparent shortcoming in that it sacrifices the strict unitarity of the second-order method. This is evident from the evaluation of the Hamiltonian at different times on each side of equation 5. However, the norm of the wavefunction never deviates significantly from unity so this formal lack of unitarity is not a problem in practice. Despite this, some may prefer to take a unitary version of this propagator by evaluating the Hamiltonian at only one point during each propagation step, for example at the midpoint of the interval.

Although this approach technically reduces the propagation from fourth-order to second-order, it is still significantly more accurate than the standard CN method. This is because the time variation of the Hamiltonian only occurs through the interaction term and is significantly slower than the time variation of the wave function, which is determined by the highest energy eigenstate occupied by the electron.

One can readily compute an upper-bound for the error arising from the unitary version of the propagator by considering plausible upper limits for the electron momentum and energy. For a Hamiltonian with a time-dependent vector potential, *A*(*t*), the difference in the wave function computed using the unitary and non-unitary expansions has a leading term in Δ*t* of

10 $$(\frac{A^{\prime} {p}_{\theta }H}{12}+\frac{iA^{\prime\prime} {p}_{\theta }}{24}){({\rm{\Delta }}t)}^{3},$$

where *A*′ and *A*′′ are the first and second derivatives of the vector potential amplitude and *p*_*θ*_ is the angular part of the momentum operator (from the diagonal part of *H*). For a slowly varying vector potential (an infrared laser field for example), this error can be small enough for this scheme to be advantageous over the CN method. There is little to be gained, however, by enforcing unitarity of the time evolution in this way. It turns out to almost always be a better option to take the unaltered fourth-order propagator, and ensure that the observable of interest is converged with respect to size of the time step.

We demonstrate that the use of this fourth order propagation scheme has advantages over the more common CN method, particularly for simulations that require high degrees of accuracy.

### The stabilized bi-conjugate gradient method

We represent the wave function Ψ(***r***) in terms of some set of basis states *ϕ*_*i*_(***r***),

11 $${\rm{\Psi }}({\bf{r}},t)=\sum _{i}\,{x}_{i}(t){\varphi }_{i}({\bf{r}}),$$

where *x*_*i*_(*t*) are the expansion coefficients.

Propagation by either equations 4 or 5 amounts to a matrix equation of the form

12 $${\bf{A}}\cdot {\bf{x}}={\bf{b}},$$

where ***b*** can be calculated directly by projection of the RHS of equations 4 or 5 onto the the basis states while the LHS can be similarly cast as a known matrix, **A**, multiplied by the coefficients of the wavefunction at *t* + Δ*t*. **A** will typically be a sparse matrix, coupling each basis element to only a handful of other elements. We solve this equation using the preconditioned BiCG-STAB^29^, a Krylov subspace method that offers smoother and faster convergence than its predecessors, the bi-conjugate gradient (BiCG) and conjugate gradient squared (CG-S) methods.

The routine converges well only if the matrix is close to the identity. To achieve this a preconditioning matrix, $\tilde{{\bf{A}}}$, is uesd that is some approximation to **A**. The preconditioned equation is then

13 $$({\bf{A}}\cdot {\tilde{{\bf{A}}}}^{-1})\cdot (\tilde{{\bf{A}}}\cdot {\bf{x}})={\bf{b}}\mathrm{.}$$

Each iteration of the algorithm requires two inversions of the preconditioning matrix, $\tilde{{\bf{A}}}$, two multiplications by **A**, and four vector inner products. The algorithm exits when the norm of the residual is less than a predefined tolerance.

### Application to atoms in intense laser fields

The electron wave function is decomposed as a partial wave expansion such that

14 $${\rm{\Psi }}({\bf{r}},t)=\sum _{l=0}\,\frac{1}{r}{\psi }_{l}(r,t){Y}_{l}^{m}(\theta ,\varphi \mathrm{).}$$

The radial functions, *ψ*_*l*_, are discretized on a linear grid. A dynamically expanding grid is used for computations of photo-electron spectra to ensure the wavefunction at the grid boundary is zero. For simulations of HHG for which ionised electrons play no role, the maximum grid size is set such that maximum electron excursion distances are entirely within the grid and a masking function is used to prevent reflections from the grid boundary.

The interaction with the laser field is performed in velocity gauge. Velocity and length gauge representations differ by a phase factor in the wavefunction. It has been shown previously that this phase factor results vastly more terms in expansion of equation 14 when length gauge is used, making length gauge computations impractical^21,28^.

In this basis, the non-discretized Hamiltonian becomes tri-diagonal in the angular quantum number, *l*, such that the laser interaction couples each *ψ*_*l*_ only to *ψ*_*l*±1_:

15 $$H{\rm{\Psi }}=(\begin{array}{ccccc}{C}_{0} & {B}_{0} &  &  & \\ {B}_{0}^{\dagger } & {C}_{1} & \ddots  &  & \\  & \ddots  & \ddots  & {B}_{l-1} & \\  &  & {B}_{l-1}^{\dagger } & {C}_{l} & \ddots \\  &  &  & \ddots  & \ddots \end{array})\,(\begin{array}{c}{\psi }_{0}\\ {\psi }_{1}\\ \vdots \\ {\psi }_{l}\\ \vdots \end{array}),$$

with

16 $${C}_{l}=-\,\frac{1}{2}\frac{{d}^{2}}{d{r}^{2}}+V(r)+\frac{l(l+1)}{2{r}^{2}}$$

17 $${B}_{l}=i{c}_{l}A(t)(\frac{d}{dr}+\frac{l+1}{r}).$$

The term *c*_*l*_ is the coupling coefficient

18 $${c}_{l}=\frac{(l+1)}{\sqrt{(2l+1)(2l+3)}}$$

and *A*(*t*) is the magnitude of the vector potential of the laser field.

Discritization is performed using a five-point finite difference approximation for the the spatial derivatives. The approximations at the *i*th grid element are given by

19 $${f^{\prime} }_{i}=\frac{{f}_{i-2}-8{f}_{i-1}+8{f}_{i+1}-{f}_{i+2}}{12h}+O({h}^{5}),$$

20 $${f^{\prime\prime} }_{i}=\frac{-{f}_{i-2}+16{f}_{i-1}-30{f}_{i}+16{f}_{i+1}-{f}_{i+2}}{12{h}^{2}}+O({h}^{5}),$$

when acting on a function *f* with a grid spacing *h*.

For a radial grid there is an inherent complication in these formulas at the first grid point *r* = *h* since the above expressions require a value at *r* = −*h* (a value at *r* = 0 is implicitly included as a result of the boundary condition at the origin). Neglecting this complication results in an error in the first grid point independent of step size.

This problem could be remedied by altering a few elements in the top left of the discritized *C*_*l*_ and *B*_*l*_ matrices, but unfortunately there is no general way of accomplishing this without destroying the hermiticity of the Hamiltonian operator.

A solution is to use an analytic expression for the radial wave functions near the origin to extrapolate to a point at *r* = −*h*. This approach is similar to that of Salomonson and Öster’s for a logarithmic grid spacing^31^. The analytic form of the radial functions is given by

21 $${\psi }_{l}(r)\propto {r}^{l+1}(1-\frac{Zr}{l+1}),$$

where *Z* is the atomic number of the atom. Close to the origin the form of the wavefunction becomes independent of energy due to the unbounded magnitude of the nuclear potential and so this approximation will hold regardless of the state of the electron.

Equation 21 indicates that radial functions are *O*(*r*^*l*+1^) as $r\to 0$ and the extrapolated point will be well approximated by zero for *l* ≥ 2. It is therefore sufficient to only consider the *l* = 0 and *l* = 1 radial functions. Extrapolated values at *r* = −*h* can be computed in terms of the value at *r* = *h*:

22 $${\psi }_{0}(\,-\,h)=-\,(1+\frac{2Zh}{1-Zh})\,{\psi }_{0}(h),$$

23 $${\psi }_{1}(\,-\,h)=(1+\frac{2Zh}{2-Zh})\,{\psi }_{1}(h).$$

Incorporating the values results in modifications to the discritized first and second derivative operators. For the second derivative,

24 $${{\rm{\Delta }}}^{(2)}=\frac{1}{12{h}^{2}}\,(\begin{array}{ccccc}a & 16 & -1 &  & \\ 16 & -30 & 16 & -1 & \\ -1 & 16 & -30 & 16 & \ddots \\  & -1 & 16 & -30 & \ddots \\  &  & \ddots  & \ddots  & \ddots \end{array}),$$

where *a* is chosen to suit the angular number of the relevant partial wave, with *a* = −29 + 2*Zh*/(1 − *Zh*) for *C*_0_ and *a* = −31 − 2*Zh*/(2 − *hZ*) for *C*_1_.

The operators *B* and *B*^†^ in equation 15 act on radial functions of different angular number so the explicit dependence of equation 21 on *l* is problematic for the hermiticity of the Hamiltonian. The second term in equation 23 is not required to obtain a sensible value for the first derivative and can be safely modified to restore hermiticity by setting *ψ*_1_(−*h*) = [1 + 2*Zh*/(1 − *Zh*)]*ψ*_1_(*h*). The discritized first derivative operator Δ^(1)^ is then

25 $${{\rm{\Delta }}}^{(1)}=\frac{1}{12h}\,(\begin{array}{ccccc}\pm b & 8 & -1 &  & \\ -8 & 0 & 8 & -1 & \\ 1 & -8 & 0 & 8 & \ddots \\  & 1 & -8 & 0 & \ddots \\  &  & \ddots  & \ddots  & \ddots \end{array}),$$

where the plus is used for the unconjugated *B*_*l*_ matrices and the minus is used for the hermitian conjugates ${B}_{l}^{\dagger }$ (this is hermitian due to the *i* that pre-multiplies the derivative in equation 17). For *B*_0_, we obtain *b* = 1 + 2*hZ*/(1 − *Zh*) and for *B*_1_ *b* = −1 + 2*hZ*/(2 − *Zh*).

No such modifications are required at the large *r* grid boundary since the wavefunction is guaranteed to be zero by the use of either a masking function or a dynamically expanding grid.

### Preconditioning

To work effectively, BiCG-STAB requires a pre-conditioning matrix, $\tilde{{\bf{A}}}$, that can be easily inverted and functions to enhance the diagonal dominance of the matrix fed to the conjugate gradient algorithm. A suitable choice for this purpose is the part of the matrix that is diagonal in *l*. For the CN propagator, this is just

26 $$\tilde{{\bf{A}}}=1+i\frac{{\rm{\Delta }}t}{2}{H}_{{\rm{at}}.},$$

while for the fourth-order propagator this is

27 $$\tilde{{\bf{A}}}=1+i\frac{{\rm{\Delta }}t}{2}{H}_{{\rm{at}}.}-\frac{{\rm{\Delta }}{t}^{2}}{12}{H}_{{\rm{at}}.}^{2},$$

where *H*_at._ is the atomic part of the Hamiltonian: *H* as defined in equation 15 without the *B*_*l*_ operators. Since the function of this matrix is simply to act as an approximation to the full matrix, we can employ a three-point finite difference representation for the spatial derivative. This ensures that the preconditioning matrix is tridiagonal in the case of the CN propagator and is easily invertible. The fourth-order preconditioner (equation 27) can be factorized into two tridiagonal matrices, which can be inverted sequentially.

This particular form of preconditioning mirrors the splitting of the propagation matrix used for the MI method, for which the part diagonal in *l* is inverted directly and the ‘off diagonal’ part is handled by the iterative routine.

### Adaptive time stepping

The numerical integration becomes much more efficient when an adaptive time step is used. At the beginning or end of the laser pulse for example, relatively large time steps can be taken compared to when the laser vector potential is large or is changing rapidly.

Both the CN and the fourth-order methods allow for embedded error checking by constructing lower-order estimates of the wave function at the next time step and using the difference between this and the higher-order value as an error estimate. This can then be used to track the error and adjust the time step accordingly. For the CN method a first-order approximation to Ψ_*n*+1_ is given by

28 $${\tilde{{\rm{\Psi }}}}_{n+1}={{\rm{\Psi }}}_{n}-i{\rm{\Delta }}tH({t}_{n}){{\rm{\Psi }}}_{n}.$$

The error estimate vector is then

29 $${\varepsilon }_{{\rm{CN}}}={{\rm{\Psi }}}_{n}-{{\rm{\Psi }}}_{n+1}-i{\rm{\Delta }}tH({t}_{n}){{\rm{\Psi }}}_{n}.$$

The error estimate for the fourth-order method is slightly more complicated to derive but ultimately reduces to

30 $${\varepsilon }_{(4)}=2{{\rm{\Psi }}}_{n}-2{{\rm{\Psi }}}_{n+1}-i{\rm{\Delta }}tH({t}_{n}){{\rm{\Psi }}}_{n}-i{\rm{\Delta }}tH({t}_{n+1}){{\rm{\Psi }}}_{n+1}.$$

Only the last term in this expression has not already been calculated in performing the propagation so computation of the error is relatively low cost.

Using this information to choose an appropriate next step size requires some trial and error. A successful method is to estimate the total error in a time step by

31 $${\varepsilon }_{{\rm{total}}}={(\sum _{i}|{\varepsilon }_{i}{|}^{2})}^{1/2},$$

where the summation ignores terms for which |Ψ|^2^ is less than a cut-off, set to 10^−5^, to ensure computational effort is not being spent where the wave function is negligible. The following time step is set so the error is less than a desired tolerance, *η*, by

32 $${\rm{\Delta }}{t}_{n+1}={\rm{\Delta }}{t}_{n}\times 0.98{(\frac{\eta }{\varepsilon })}^{\mu },$$

The exponent *μ* is set according to the order of error of the time step — 0.2 for the fourth-order method and 0.33 for Crank-Nicolson.

While this adaptive stepping scheme produces converged results for both the CN and fourth-order propagators, we find that it works best for the fourth-order method. This is likely because the estimate of the error is closer to the true error in this case.

## Results

### Convergence of the CG algorithm

To test the performance of the BiCG-STAB routine we compare directly with the MI method. As remarked earlier the splitting of the workload of the two schemes is very similar with both involving direct inversion of the part of the matrix that is diagonal in the orbital quantum number *l* — in the BiCG-STAB scheme this is done through the preconditioner — while parts off-diagonal in *l* are handled by the iterative scheme. We can therefore meaningfully compare the two schemes iteration-by-iteration.

**Figure 1:**
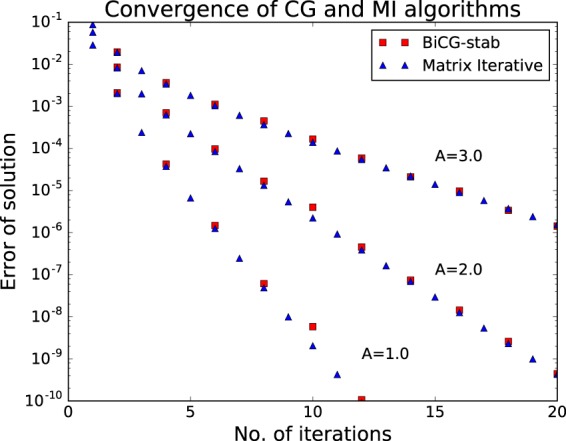
Convergence of Matrix Iterative (MI) and Conjugate Gradient (CG) routines. The plot shows the similarity of the convergence characteristics of the two algorithms. For the sake of comparison with MI, one iteration of the BiCG-STAG algorithm is considered as two iterations. Identical matrix equations are being solved in each case (i.e. with a three-point finite difference formula).

The calculations simulate a hydrogen atom initially in its ground state. The matrix equation we use as a test case corresponds to a single time step of 0.1 atomic units in the presence of a vector potential. The magnitude of the vector potential is set to values of 1.0, 2.0 and 3.0 atomic units, which are typical of intense laser pulses. For the purposes of this comparison, a single iteration of the CG algorithm is counted as two iterations as it involves two updates of the solution vector and two inversions of the preconditioning matrix, meaning a single ‘iteration’ involves similar computational work for each of the methods. To facilitate a direct comparison the three-point difference formula is used in for the CG method rather than the five-point formula as defined in equations 19 and 20. When compared iteration by iteration in this fashion, the CG method is somewhat slower than MI. This is to be expected due to the bookkeeping overheads and the need to compute a search direction at each step. The convergence as a function of iteration is shown in Fig. 1.

It is noteworthy that the convergence of the two methods is almost identical. This reflects the diagonal dominance of the matrix to be inverted and the similarities in the treatment of the diagonal part of the matrix. Similar calculations were performed with the wave function in an evolved state after partial ionization by a laser pulse, but the similarities in the convergence are maintained.

**Figure 2:**
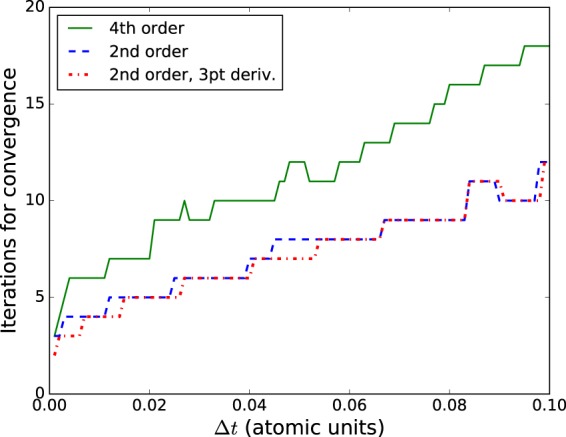
Iterations for convergence of conjugate gradient algorithm as a function of time step. Results are for evolving a wave function one time step midway through a few cycle pulse. Convergence is defined to be a residual for the matrix equation of magnitude less than 10^−10^. Results are shown for the fourth-order and second-order propagators with five-point spatial derivatives and for the second-order method with a three-point spatial derivative.

These results demonstrate no immediate advantage in using the more complex CG scheme over the MI scheme if the CN propagator and three-point methods are used. We find, however, that the convergence of the conjugate gradient routine remains strong when higher-order methods are used for the spatial and temporal derivatives. The cost of these higher-order approximations can be measured by the number of iterations required for the algorithm to converge and comparing this convergence using the lower-order methods. We expect the lower-order methods to converge more quickly due to the simplicity of the equations. Employing a five-point formula results in an insignificant additional convergence cost. The fourth-order time propagator, on the other hand, always requires more iterations to converge than its second-order counterpart, but this cost is counterbalanced by the significant reduction in required number of time steps. An example of the number of iterations required for convergence as a function of the time step size, Δ*t*, is shown in Fig. 2.

### Comparison of 3 point and 5 point difference formulas

**Figure 3:**
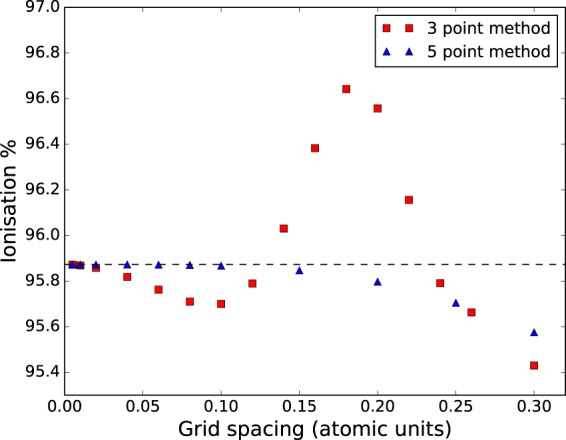
Calculated ionisation probabilities as a function of grid spacing. The five-point finite difference (triangles) results is significantly improved convergence compared to the three-point formula (squares). Laser parameters: peak intensity 10^15^ W/cm^2^, wavelength 800 nm, FWHM duration 5.0 fs.

A five-point difference method has a clear advantage over the three-point difference method. Figure 3 demonstrates how calculations of the total ionization probability depend on grid spacing for the respective methods. The simulations are of a hydrogen atom in a 5 fs laser pulse with peak intensity 10^15^ W/cm^2^.

An upper limit is imposed on the maximum grid spacing by the uncertainty principle and it is important to ensure this limit is not exceeded. Specifically, for a maximum electron momentum *p*_max_, this requirement is Δ*r* < 2*π*/*p*_max_. *p*_max_ can be estimated by considering the maximum momentum an electron could attain in the evolving laser field from a stationary start. In the simulations presented in Fig. 3 this limit is approximately 1 atomic unit, safely exceeding the maximum grid spacing used in the simulations.

For the five-point method, even with a grid spacing of 0.2 a.u., the error in the calculated ionization probability is less than 0.1% (percentage relative to the converged probability, 0.958) of the converged value. To achieve the same accuracy with the three-point method requires four times as many grid points. The corresponding benefit to computational time is not quite a factor of four, since slightly more work is done using a five-point routine (multiplication by the 5-point Hamiltonian is roughly a factor of 1.67 more expensive – 15 non-zero diagonal bands instead of 9), but the decrease in computational time is significantly better than a factor of two. As shown in Fig. 2, the number of iterations required for a time step to converge is only slightly greater, or the same, for when the five-point difference method is used, making this option clearly superior.

### Unitarity of fourth-order propagator

**Figure 4:**
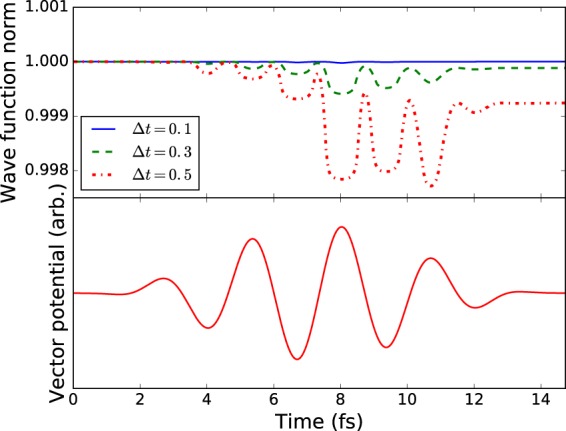
Top: The norm of the wave function when the non-unitary forth-order time propagator is used with step sizes of 0.1, 0.3 and 0.5 atomic units. Bottom: The vector potential of the laser pulse. As might be predicted, the norm changes most rapidly when the vector potential is varying the fastest since this is when the value of the Hamiltonian on the two sides of the propagation equation will be the most different.

The asymmetry of the fourth-order propagation equation (equation 5) has the potential to result in non-unitary time evolution. This is a direct result of the explicit time variation of the Hamiltonian and so complete unitarity of the propagation will be restored in the limit ${\rm{\Delta }}t\to 0$ when the laser field becomes zero. To illustrate the deviation of the norm of the wave function, Fig. 4 shows the norm plotted as a function of pulse time for a range of step sizes (which are held constant throughout the calculation). For this simulation the peak pulse intensity is set to 5 × 10^14^ W/cm^2^ with central wavelength of 800 nm and four cycles duration.

The figure shows the most rapid variation in the norm when the vector potential of the laser field is changing most rapidly, corresponding to the times when the Hamiltonian is also changing most rapidly. For Δ*t* = 0.1 a.u., this variation is negligible; significantly less than one part in ten thousand. For Δ*t* = 0.5 a.u. the maximum variation is around 0.2 percent. The clear dependence of the deviation from unit norm on the rate of change of the vector potential suggests a varying time-step would be beneficial. While it is certainly possible to use the variation of the norm of the wavefunction directly as a control parameter for the step size, the methods of adaptive step sizing described above are preferable as they more directly relate step size to the error in the propagation equations.

### Comparison of 2nd order and 4th order propagators

To compare the performance of the second-order and fourth-order propagators we examined high harmonic spectra computed using fixed time steps. We find there is a significant reduction in the number of time steps required to achieve a converged solution when the higher-order method is used – we obtain a well converged spectrum for the fourth-order method using a time step of 0.2 atomic units. To achieve similar convergence with the Crank-Nicolson method the required time step is 0.02 atomic units.

We define a metric to determine the error in a given spectrum based on a normalized difference of the spectrum to the converged spectrum (obtained using the highest accuracy simulation):

33 $${\rm{\Delta }}(\omega )=\frac{I(\omega )-{I}_{0}(\omega )}{{\bar{I}}_{0}(\omega )}$$

where ${\bar{I}}_{0}(\omega )$ is a weighted average of the spectrum at *ω* determined by

34 $${\bar{I}}_{0}(\omega )=\frac{{\int }_{\omega -\delta }^{\omega +\delta }\,{I}_{0}(\omega ^{\prime} )g(\omega ^{\prime} -\omega )d\omega ^{\prime} }{{\int }_{-\delta }^{\delta }\,g(\omega ^{\prime} )d\omega ^{\prime} }.$$

*g*(*ω*) is a windowing function with a window size 2*δ* defined by

35 $$g(\omega )={(1-|\omega {|}^{3}/{\delta }^{3})}^{3}.$$

We set *δ* to be 4 times the laser frequency.

**Figure 5:**
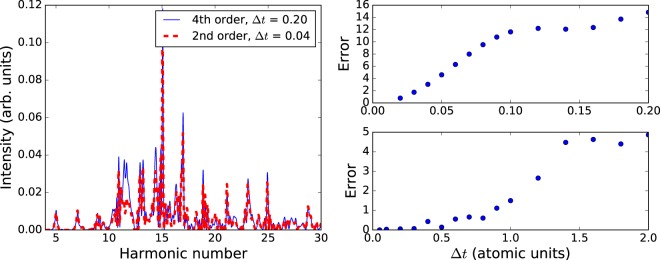
Left: Plot of harmonic spectrum for a 1400 nm laser pulse with peak intensity 2 × 10^14^ W/cm^2^ and FWHM duration 20 fs. The red line uses the fourth-order propagator with time step 0.20 a.u. and blue line uses the second-order propagator with time step 0.04 a.u. The plot of the fourth-order result is indistinguishable from the converged result indicating that a substantial increase in accuracy is obtained with the fourth-order result even for a significantly longer time step. Right: Error as defined in equation 33 integrated over *ω* for second-order (top) and fourth-order (bottom) methods as a function of time step.

In Fig. 5, we show spectra produced using the two methods. The fully converged spectrum in not shown in this plot as it is indistinguishable from the fourth-order result. The second-order method, despite having a time step five times shorter than the fourth-order method, is clearly different from the converged spectrum and is unsatisfactory for quantitative purposes. The right-hand plots of Fig. 5 show the integrated error of the harmonic spectra as defined in equation 33. This shows markedly faster convergence for the higher-order method. Importantly, both the second- and fourth-order methods converge to the same result as the time step is decreased. This indicates that the small deviation from unit norm that arises in the fourth-order method has no appreciable effect on the calculation of observables.

The cost of the higher-order method is its increased complexity, which results in more iterations per time step (see Fig. 2) and more computation at each iteration. To determine the overall performance of the higher-order method relative to the second-order method we count the total number of iterations throughout a simulation. Table 1 demonstrates how a specific harmonic intensity converges with respect to the size of time step used in the calculation. Harmonic intensity is calculated by integrating the spectrum, *I*(*ω*), over the harmonic frequency.

**Table 1:** Computed harmonic intensities and required time steps and total iterations for simulations of an atom in a moderate laser field using different methods.

Order	Δ*t*	15^th^ harm.(arb)	steps (×10^3^)	iterations (×10^3^)
2^nd^	0.01	2.47	211	626
2^nd^	0.02	2.41	105	345
2^nd^	0.04	2.16	52.8	191
2^nd^	0.10	1.17	21.1	107
4^th^	0.10	2.49	21.1	160
4^th^	0.20	2.49	11.7	127
4^th^	1.0	2.47	5.93	105
4^th^	2.0	2.26	5.43	103

Laser parameters: peak intensity 2 × 10^14^ W/cm^2^, wavelength 800 nm, FWHM duration 5.0 fs.

Once the number of required iterations exceeds 20, there appears to be no computational advantage in increasing the time step regardless of numerical accuracy, and a smaller step size results in a faster computation. To allow for this, for these simulations, whenever the number of iterations required is 20 or greater, the time step is reduced so that this limit is not exceeded. The time step is otherwise kept constant at the specified value. The only simulations for which the number of iterations per step approaches 20 at any point are the fourth-order simulations with step sizes of 0.20, 1.0 and 2.0.

The total number of iterations aggregated across the simulation are shown as an alternative measure of the time taken for each method, that is (relatively) independent of factors such as programming language and style.

Computationally, the difference between the second- and fourth-order methods stems from the fact that the number of Hamiltonian multiplications increases from two to six each iteration, while the number of inversions of the field free Hamiltonian increases from two to four. The number of dot products remains the same, although the number of vector additions increases slightly. Overall, a single iteration of the fourth-order scheme consistently takes about 1.5 times the time taken by the second-order method. Thus, the fourth-order scheme will be more efficient if the simulation can be completed with fewer than two thirds of the number for the second-order scheme. The table shows that, in calculating the 15th harmonic to within 5% accuracy, the second-order method takes 300 × 10^3^ iterations compared to approximately 105 × 10^3^ iterations for the fourth-order method. The overall time saving is around a factor of two.

In general it will be desirable to use a varying time step rather than a fixed time step, so this factor of two does not necessarily reflect the actual reduction in computation of the higher order method. The fourth order method is most advantageous when the magnitude of the vector potential is large, and high accuracy is required. Note that when the magnitude is large the variation is slow so this is also the point at which the deviation from unit norm will be smallest. In many cases, the most efficient method may be a hybrid of the second- and fourth-order schemes where the second-order method can skip through the ‘easy’ periods where the vector potential is small, taking large steps, and the fourth-order method can step in to cover the rockier terrain when the magnitude of the vector potential is large.

## Conclusion

We have presented a method of integrating the time-dependent Schrödinger equation for a single particle with a time-dependent Hamiltonian that is based around use of the stabilized bi-conjugate gradient method (BiCG-STAB) to solve a matrix equation for the propagation of a single time step. We have applied our method to the problem of an atom in an intense few-cycle infra-red laser field and demonstrated that it performs better than existing methods. Specifically, we have demonstrated that this method can incorporate higher-order approximations to spatial and temporal derivatives than other more rigid methods, and this allows for the use of significantly fewer spatial and temporal grid points without compromising on accuracy. We have shown that this reduction in grid points means a significant decrease in the amount of time required to perform simulations.
